# Poly[(μ-5,7-dihydr­oxy-4-oxo-2-phenyl-4*H*-chromene-8-sulfonato)potassium(I)]

**DOI:** 10.1107/S1600536808041779

**Published:** 2008-12-13

**Authors:** Bin Liu, Bo-Lun Yang

**Affiliations:** a Department of Chemical Engineering, Xi’an Jiaotong University, Xi’an 710049, People’s Republic of China; b Department of Chemistry, Xianyang Normal University, Xianyang 712000, People’s Republic of China

## Abstract

In the polymeric title compound, [K(C_15_H_9_O_7_S)]_*n*_, the potassium cation is five-coordinated by four sulfonate O atoms and one carbonyl O atom. Two intra­molecular O—H⋯O hydrogen bonds stabilize the conformation of the anion. The polymeric three-dimensional supra­molecular architecture is formed *via* coordination inter­actions and π–π stacking inter­actions involving centrosymmetrically related pyrone rings, with a centroid–centroid separation of 3.513 (2) Å.

## Related literature

For biological activities of flavonoids, see: Aljancic *et al.* (1999 ▶); Habtemariam (1997 ▶); Knekt *et al.* (1997 ▶); Ko *et al.* (1998 ▶); Nkengfack *et al.* (1994 ▶); Sakaguchi *et al.* (1992 ▶). For related structures, see: Benedict *et al.* (2004 ▶); Li & Zhang (2008 ▶); Wang & Zhang (2005*a* ▶,*b* ▶); Zhang & Wang (2005*a* ▶,*b* ▶).
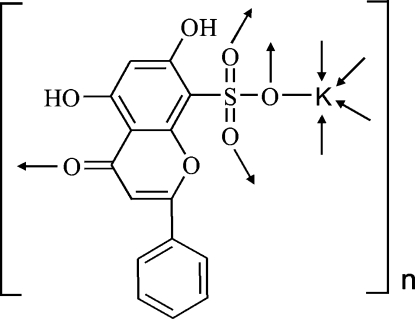


## Experimental

### 

#### Crystal data

[K(C_15_H_9_O_7_S)]
*M* *_r_* = 372.38
Orthorhombic, 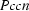

*a* = 19.0846 (19) Å
*b* = 20.6555 (19) Å
*c* = 7.5148 (7) Å
*V* = 2962.3 (5) Å^3^
*Z* = 8
Mo *K*α radiation
μ = 0.54 mm^−1^
*T* = 296 (2) K
0.37 × 0.20 × 0.13 mm

#### Data collection

Bruker SMART-1000 CCD area-detector diffractometer
Absorption correction: multi-scan (*SADABS*; Bruker, 1999 ▶) *T*_min_ = 0.825, *T*_max_ = 0.933
13998 measured reflections
2637 independent reflections
1746 reflections with *I* > 2σ(*I*)
*R*_int_ = 0.049

#### Refinement

*R*[*F* ^2^ > 2σ(*F* ^2^)] = 0.044
*wR*(*F* ^2^) = 0.152
*S* = 1.02
2637 reflections
220 parameters
H-atom parameters constrained
Δρ_max_ = 0.26 e Å^−3^
Δρ_min_ = −0.29 e Å^−3^

### 

Data collection: *SMART* (Bruker, 1999 ▶); cell refinement: *SAINT-Plus* (Bruker, 1999 ▶); data reduction: *SAINT-Plus*; program(s) used to solve structure: *SHELXS97* (Sheldrick, 2008 ▶); program(s) used to refine structure: *SHELXL97* (Sheldrick, 2008 ▶); molecular graphics: *SHELXTL* (Sheldrick, 2008 ▶); software used to prepare material for publication: *SHELXTL*.

## Supplementary Material

Crystal structure: contains datablocks I, global. DOI: 10.1107/S1600536808041779/rz2268sup1.cif

Structure factors: contains datablocks I. DOI: 10.1107/S1600536808041779/rz2268Isup2.hkl

Additional supplementary materials:  crystallographic information; 3D view; checkCIF report

## Figures and Tables

**Table 1:** Selected bond lengths (Å)

K1—O5^i^	2.674 (2)
K1—O6^ii^	2.754 (2)
K1—O7^iii^	2.655 (3)
K1—O3^iv^	2.642 (2)
K1—O6	2.756 (3)

Symmetry codes: (i) 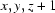; (ii) 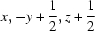; (iii) 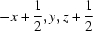; (iv) 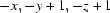.

**Table 2:** Hydrogen-bond geometry (Å, °)

*D*—H⋯*A*	*D*—H	H⋯*A*	*D*⋯*A*	*D*—H⋯*A*
O1—H1⋯O7	0.82	1.84	2.588 (4)	152
O2—H2⋯O3	0.82	1.87	2.604 (4)	148

Symmetry codes: .

**Fig. 1.**
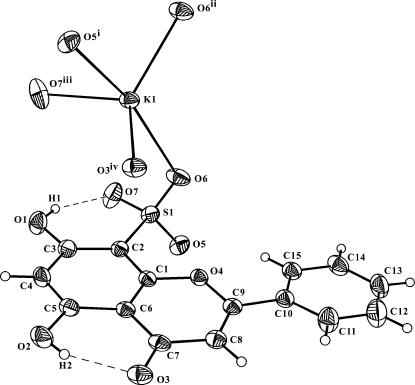
The coordination environment of the potassium(I) cation in the title compound, showing the atom-numbering scheme. Displacement ellipsoids are drawn at the 30% probability level. Intramolecular hydrogen bonds are shown as dashed lines. Symmetry codes: (i) x, y, z + 1; (ii) x, -y + 1/2, z + 1/2; (iii) -x + 1/2, y, z + 1/2; (iv) -x, -y + 1, -z + 1.

**Fig. 2.**
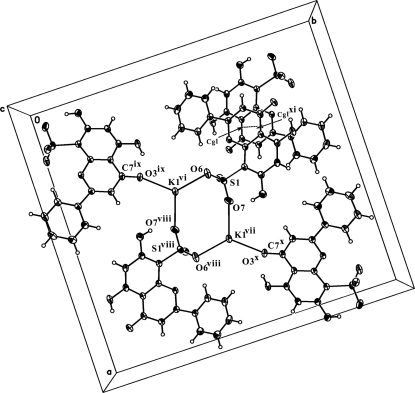
Packing diagram of the title compound viewed along the c axis, showing the sheet-like structure in the ab plane. The dashed line indicates the π–π stacking interaction. Cg1 is the centroid of the pyrone ring. Symmetry codes: (vi) x, -y + 1/2, z - 1/2; (vii) -x + 1/2, y, z - 1/2; (viii) -x + 1/2, -y + 1/2, z; (ix) -x, y - 1/2, -z + 1/2; (x) x + 1/2, -y + 1, -z + 1/2; (xi) -x, -y + 1, -z.

**Fig. 3.**
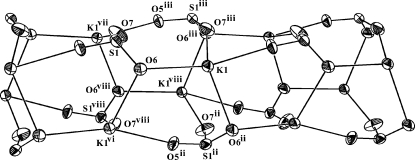
The one-dimensional polyion chain along the c axis in the title compound. Symmetry codes: (ii) x, -y + 1/2, z + 1/2; (iii) -x + 1/2, y, z + 1/2; (vi) x, -y + 1/2, z - 1/2; (vii) -y + 1/2, y, z - 1/2; (viii) -x + 1/2, -y + 1/2, z.

## supplementary crystallographic information

### Comment

The biological properties of flavonoids (2-phenylbenzo-γ-pyrone derivatives) are well known (Sakaguchi *et al.*, 1992; Nkengfack *et al.*, 1994; Habtemariam, 1997; Knekt *et al.*, 1997; Ko *et al.*, 1998; Aljancic *et al.*, 1999). The main problem that these compounds present for their use in biological experiments is their poor solubility in water. To overcome this problem, many flavonoidsulfonate derivatives have been synthesized, for example [Ni(H_2_O)_6_](C_17_H_13_O_7_S)_2_.8H_2_O (Zhang & Wang, 2005*a*), [Sr(H_2_O)_7_(C_16_H_11_O_4_SO_3_)][C_16_H_11_O_4_SO_3_].6H_2_O (Zhang & Wang, 2005*b*), [Mg(H_2_O)_6_](C_19_H_17_O_4_SO_3_)_2_].8H_2_O and [Zn(H_2_O)_6_](C_19_H_17_O_4_SO_3_)_2_].8H_2_O (Wang & Zhang, 2005*a*) and [Co(H_2_O)_6_](C_19_H_17_O_4_SO_3_)_2_.8H_2_O (Wang & Zhang, 2005*b*). Against this background, we report here the solid-state characterization of the potassium salt of 5,7-dihydroxyflavone-8-sulfonate.

The asymmetric unit of title compound consists of one potassium(I) cation and one 5,7-dihydroxyflavone-8-sulfonate anion (Fig. 1). The cation has a distorted coordination geometry and is coordinated by four sulfonate O atoms (O5^i^, O6, O6^ii^, and O7^iii^; symmetry codes: (i) *x*, *y*, *z* + 1; (ii) *x*, -*y* + 1/2, *z* + 1/2; (iii) -*x* + 1/2, *y*, *z* + 1/2) and one carbonyl O atom (O3^iv^; symmetry code: (iv) -*x*, -*y* + 1, -*z* + 1). The K—O bond distances (Table 1) are in agreement with those found in K^+^.C_16_H_11_O_8_^-^.2H_2_O (Benedict *et al.*, 2004). The flavone skeleton presents the same structure of a corresponding flavonesulfonate ligand reported previously (Li & Zhang, 2008) and is stabilized by intramolecular hydrogen bonds, with O1—H1···O7 = 2.588 (4) Å and O2—H2···O3 = 2.640 (4) Å (Table 2).

As shown in Fig. 2, K1^vi^ (symmetry code: (vi) *x*, -*y* + 1/2, *z* - 1/2) and K1^vii^ (symmetry code: (vii) -*x* + 1/2, *y*, *z* - 1/2) coordinate with O6 and O7^viii^ (symmetry code: (viii) -*x* + 1/2, -*y* + 1/2, *z*) and O6^viii^ and O7, respectively, resulting in a centrosymmetric eight-membered chelate ring that is non-planar. Coordination bonds K1^vi^–O3^ix^ (symmetry code: (ix) -*x*, *y* - 1/2, -*z* + 1/2) and K1^vii^–O3^x^ (symmetry code: (*x*) *x* + 1/2, -*y* + 1, -*z* + 1/2) link the flavone skeletons [C1—C15/O4]^ix^ and [C1—C15/O4]^x^ and the eight-membered ring together to form a two-dimensional sheet-like structure in the *ab* plane. Another eight-membered chelate ring is built up by O6^ii^, S1^ii^, O7^ii^, K1^viii^, O6^iii^, S1^iii^, O7^iii^ and K1. Two adjacent eight-membered rings are further linked into a one-dimensional polyion chain along the *c* axis by the coordination bonds K1–O6, K1^vii^–O5^iii^, K1^viii^–O6^viii^ and K1^vi^–O5^ii^ (Fig. 3). Additionally, π···π stacking interactions (Fig. 2) between centrosymmetrically related pyrone rings may be effective in the stabilization of the three-dimensional polymeric structure, with a centroid-centroid distance of 3.513 (2) Å, a perpendicular interplanar distance of 3.342 (3) Å and a centroid···centroid offset of 1.084 (2) Å.

### Experimental

5,7-dihydroxyflavone (1.0 g, 3.9 mmol) was added slowly to concentrated sulfuric acid (6 ml) with stirring. The reaction was maintained at room temperature for 12 h. Then, the mixture was poured into a KCl saturated aqueous solution (50 ml) and a yellow precipitate appeared. After 5 h, the precipitate was filtered and washed with a KCl saturated aqueous solution until the pH value of the filtrate was 7. The solid product was recrystallized from an ethanol-water (1:1 *v/v*) solution. Colourless plate-shaped crystals suitable for X-ray analysis were obtained by slow evaporation of the solvent for about 4 d at room temperature (yield 83%).

### Refinement

All H atoms were positioned geometrically and refined using a riding model, with C—H = 0.93 Å, O–H = 0.82 Å, and with *U*_iso_(H) = 1.2 *U*_eq_(C) or 1.5 *U*_eq_(O).

### Crystal data

**Table:**

[K(C_15_H_9_O_7_S)]	*F*(000) = 1520
*M**_r_* = 372.38	*D*_x_ = 1.670 Mg m^−^^3^
Orthorhombic, *P**c**c**n*	Mo *K*α radiation, λ = 0.71073 Å
Hall symbol: -P 2ab 2ac	Cell parameters from 2056 reflections
*a* = 19.0846 (19) Å	θ = 2.9–20.9°
*b* = 20.6555 (19) Å	µ = 0.54 mm^−^^1^
*c* = 7.5148 (7) Å	*T* = 296 K
*V* = 2962.3 (5) Å^3^	Plate, colourless
*Z* = 8	0.37 × 0.20 × 0.13 mm

### Data collection

**Table:**

Bruker SMART-1000 CCD area-detector diffractometer	2637 independent reflections
Radiation source: fine-focus sealed tube	1746 reflections with *I* > 2σ(*I*)
graphite	*R*_int_ = 0.049
φ and ω scans	θ_max_ = 25.1°, θ_min_ = 1.5°
Absorption correction: multi-scan (*SADABS*; Bruker, 1999)	*h* = −21→22
*T*_min_ = 0.825, *T*_max_ = 0.933	*k* = −24→22
13998 measured reflections	*l* = −7→8

### Refinement

**Table:**

Refinement on *F*^2^	Secondary atom site location: difference Fourier map
Least-squares matrix: full	Hydrogen site location: inferred from neighbouring sites
*R*[*F*^2^ > 2σ(*F*^2^)] = 0.044	H-atom parameters constrained
*wR*(*F*^2^) = 0.152	*w* = 1/[σ^2^(*F*_o_^2^) + (0.091*P*)^2^] where *P* = (*F*_o_^2^ + 2*F*_c_^2^)/3
*S* = 1.02	(Δ/σ)_max_ < 0.001
2637 reflections	Δρ_max_ = 0.26 e Å^−^^3^
220 parameters	Δρ_min_ = −0.28 e Å^−^^3^
0 restraints	Extinction correction: *SHELXL97* (Sheldrick, 2008), Fc^*^=kFc[1+0.001xFc^2^λ^3^/sin(2θ)]^-1/4^
Primary atom site location: structure-invariant direct methods	Extinction coefficient: none

### Special details

**Table:**

Geometry. All e.s.d.'s (except the e.s.d. in the dihedral angle between two l.s. planes) are estimated using the full covariance matrix. The cell e.s.d.'s are taken into account individually in the estimation of e.s.d.'s in distances, angles and torsion angles; correlations between e.s.d.'s in cell parameters are only used when they are defined by crystal symmetry. An approximate (isotropic) treatment of cell e.s.d.'s is used for estimating e.s.d.'s involving l.s. planes.
Refinement. Refinement of *F*^2^ against ALL reflections. The weighted *R*-factor *wR* and goodness of fit *S* are based on *F*^2^, conventional *R*-factors *R* are based on *F*, with *F* set to zero for negative *F*^2^. The threshold expression of *F*^2^ > σ(*F*^2^) is used only for calculating *R*-factors(gt) *etc*. and is not relevant to the choice of reflections for refinement. *R*-factors based on *F*^2^ are statistically about twice as large as those based on *F*, and *R*- factors based on ALL data will be even larger.

### Fractional atomic coordinates and isotropic or equivalent isotropic displacement parameters (Å^2^)

**Table:**

	*x*	*y*	*z*	*U*_iso_*/*U*_eq_	
K1	0.13853 (4)	0.30967 (3)	0.70530 (10)	0.0614 (3)	
S1	0.15766 (5)	0.35454 (4)	0.23290 (12)	0.0609 (3)	
O1	0.24816 (13)	0.46137 (12)	0.4226 (4)	0.0808 (8)	
H1	0.2561	0.4241	0.3913	0.121*	
O2	0.07320 (14)	0.61774 (10)	0.4594 (4)	0.0757 (8)	
H2	0.0318	0.6235	0.4351	0.114*	
O3	−0.04849 (13)	0.59352 (11)	0.3174 (3)	0.0681 (7)	
O4	0.02313 (11)	0.41527 (10)	0.1793 (3)	0.0518 (6)	
O5	0.14317 (13)	0.34558 (12)	0.0469 (3)	0.0711 (7)	
O6	0.11604 (17)	0.31214 (11)	0.3432 (3)	0.0830 (8)	
O7	0.23185 (16)	0.34965 (14)	0.2716 (4)	0.1005 (11)	
C1	0.06796 (17)	0.45769 (15)	0.2611 (4)	0.0483 (8)	
C2	0.13557 (17)	0.43436 (15)	0.2928 (4)	0.0510 (8)	
C3	0.18152 (19)	0.47689 (16)	0.3815 (4)	0.0587 (8)	
C4	0.15971 (19)	0.53813 (16)	0.4343 (5)	0.0637 (9)	
H4	0.1910	0.5653	0.4928	0.076*	
C5	0.09334 (19)	0.55884 (15)	0.4016 (4)	0.0574 (8)	
C6	0.04475 (17)	0.51880 (14)	0.3107 (4)	0.0494 (8)	
C7	−0.02559 (19)	0.53857 (15)	0.2717 (4)	0.0555 (9)	
C8	−0.06835 (18)	0.49192 (16)	0.1810 (4)	0.0563 (8)	
H8	−0.1140	0.5029	0.1495	0.068*	
C9	−0.04401 (17)	0.43304 (15)	0.1409 (4)	0.0515 (8)	
C10	−0.08112 (18)	0.38009 (16)	0.0517 (4)	0.0571 (8)	
C11	−0.1517 (2)	0.3838 (2)	0.0188 (6)	0.0862 (13)	
H11	−0.1761	0.4205	0.0549	0.103*	
C12	−0.1872 (3)	0.3350 (2)	−0.0658 (8)	0.1055 (16)	
H12	−0.2352	0.3384	−0.0857	0.127*	
C13	−0.1515 (3)	0.2812 (2)	−0.1205 (6)	0.0930 (14)	
H13	−0.1751	0.2485	−0.1806	0.112*	
C14	−0.0811 (3)	0.27500 (18)	−0.0878 (6)	0.0805 (12)	
H14	−0.0572	0.2380	−0.1237	0.097*	
C15	−0.0457 (2)	0.32438 (16)	−0.0007 (4)	0.0654 (9)	
H15	0.0019	0.3202	0.0229	0.079*	

### Atomic displacement parameters (Å^2^)

**Table:**

	*U*^11^	*U*^22^	*U*^33^	*U*^12^	*U*^13^	*U*^23^
K1	0.0902 (6)	0.0465 (5)	0.0474 (5)	0.0031 (3)	−0.0002 (4)	−0.0003 (3)
S1	0.0824 (7)	0.0503 (5)	0.0499 (6)	0.0108 (4)	−0.0088 (4)	−0.0111 (4)
O1	0.0802 (18)	0.0717 (17)	0.0906 (19)	0.0055 (13)	−0.0169 (15)	−0.0260 (15)
O2	0.110 (2)	0.0437 (13)	0.0731 (18)	0.0056 (13)	−0.0029 (16)	−0.0139 (12)
O3	0.0923 (18)	0.0503 (14)	0.0618 (15)	0.0184 (12)	0.0040 (12)	−0.0021 (11)
O4	0.0652 (14)	0.0425 (12)	0.0477 (13)	−0.0009 (10)	0.0000 (10)	−0.0024 (10)
O5	0.0899 (17)	0.0801 (17)	0.0432 (15)	0.0097 (13)	−0.0021 (11)	−0.0177 (12)
O6	0.144 (2)	0.0461 (14)	0.0583 (15)	0.0019 (14)	0.0097 (16)	0.0017 (12)
O7	0.089 (2)	0.089 (2)	0.124 (3)	0.0313 (16)	−0.0417 (18)	−0.0468 (17)
C1	0.065 (2)	0.0459 (18)	0.0340 (17)	−0.0041 (14)	0.0040 (14)	0.0003 (13)
C2	0.068 (2)	0.0476 (18)	0.0368 (17)	0.0016 (15)	−0.0004 (15)	−0.0012 (14)
C3	0.074 (2)	0.0527 (19)	0.050 (2)	−0.0003 (17)	−0.0002 (17)	−0.0027 (16)
C4	0.082 (3)	0.050 (2)	0.059 (2)	−0.0092 (17)	−0.0038 (18)	−0.0100 (17)
C5	0.084 (2)	0.0445 (18)	0.0434 (19)	−0.0014 (16)	0.0025 (17)	−0.0013 (15)
C6	0.069 (2)	0.0425 (17)	0.0366 (17)	0.0021 (15)	0.0032 (15)	0.0009 (13)
C7	0.083 (2)	0.0437 (18)	0.0395 (17)	0.0045 (16)	0.0111 (16)	0.0056 (14)
C8	0.067 (2)	0.055 (2)	0.047 (2)	0.0026 (16)	0.0066 (16)	0.0026 (15)
C9	0.063 (2)	0.0546 (19)	0.0368 (17)	−0.0023 (16)	0.0067 (15)	0.0058 (14)
C10	0.067 (2)	0.056 (2)	0.048 (2)	−0.0070 (16)	0.0021 (16)	0.0012 (16)
C11	0.080 (3)	0.080 (3)	0.099 (3)	−0.005 (2)	−0.006 (2)	−0.021 (3)
C12	0.081 (3)	0.096 (3)	0.139 (5)	−0.013 (3)	−0.012 (3)	−0.021 (3)
C13	0.106 (4)	0.088 (3)	0.085 (3)	−0.039 (3)	−0.015 (3)	−0.006 (3)
C14	0.116 (4)	0.057 (2)	0.069 (3)	−0.015 (2)	0.002 (2)	−0.0078 (19)
C15	0.085 (3)	0.059 (2)	0.052 (2)	−0.0076 (19)	−0.0020 (18)	0.0029 (17)

### Geometric parameters (Å, °)

**Table:**

K1—O5^i^	2.674 (2)	C1—C2	1.398 (4)
K1—O6^ii^	2.754 (2)	C2—C3	1.409 (5)
K1—O7^iii^	2.655 (3)	C3—C4	1.389 (5)
K1—O3^iv^	2.642 (2)	C4—C5	1.359 (5)
K1—O6	2.756 (3)	C4—H4	0.9300
K1—S1^ii^	3.4177 (12)	C5—C6	1.418 (4)
S1—O5	1.437 (2)	C6—C7	1.433 (5)
S1—O6	1.444 (3)	C7—C8	1.435 (5)
S1—O7	1.449 (3)	C8—C9	1.336 (4)
S1—C2	1.760 (3)	C8—H8	0.9300
O1—C3	1.347 (4)	C9—C10	1.466 (4)
O1—H1	0.8200	C10—C11	1.371 (5)
O2—C5	1.348 (4)	C10—C15	1.391 (5)
O2—H2	0.8200	C11—C12	1.372 (6)
O3—C7	1.264 (4)	C11—H11	0.9300
O3—K1^iv^	2.642 (2)	C12—C13	1.366 (6)
O4—C9	1.364 (4)	C12—H12	0.9300
O4—C1	1.370 (4)	C13—C14	1.372 (6)
O5—K1^v^	2.674 (2)	C13—H13	0.9300
O6—K1^vi^	2.754 (2)	C14—C15	1.387 (5)
O7—K1^vii^	2.655 (3)	C14—H14	0.9300
C1—C6	1.389 (4)	C15—H15	0.9300
			
O3^iv^—K1—O7^iii^	112.50 (9)	C5—O2—H2	109.5
O3^iv^—K1—O5^i^	82.73 (7)	C7—O3—K1^iv^	152.5 (2)
O7^iii^—K1—O5^i^	72.70 (8)	C9—O4—C1	120.6 (2)
O3^iv^—K1—O6^ii^	127.89 (9)	S1—O5—K1^v^	167.47 (15)
O7^iii^—K1—O6^ii^	111.02 (10)	S1—O5—K1^vi^	77.73 (11)
O5^i^—K1—O6^ii^	84.11 (8)	K1^v^—O5—K1^vi^	94.19 (7)
O3^iv^—K1—O6	79.69 (8)	S1—O6—K1^vi^	104.63 (13)
O7^iii^—K1—O6	108.95 (10)	S1—O6—K1	119.49 (16)
O5^i^—K1—O6	161.50 (8)	K1^vi^—O6—K1	109.29 (8)
O6^ii^—K1—O6	111.35 (7)	S1—O7—K1^vii^	153.43 (16)
O3^iv^—K1—S1^ii^	145.54 (6)	O4—C1—C6	120.2 (3)
O7^iii^—K1—S1^ii^	101.41 (7)	O4—C1—C2	115.6 (3)
O5^i^—K1—S1^ii^	102.33 (6)	C6—C1—C2	124.2 (3)
O6^ii^—K1—S1^ii^	24.13 (6)	C1—C2—C3	116.1 (3)
O6—K1—S1^ii^	95.45 (5)	C1—C2—S1	120.0 (2)
O3^iv^—K1—O5^ii^	134.72 (7)	C3—C2—S1	123.8 (3)
O7^iii^—K1—O5^ii^	109.43 (8)	O1—C3—C4	115.7 (3)
O5^i^—K1—O5^ii^	126.38 (7)	O1—C3—C2	123.2 (3)
O6^ii^—K1—O5^ii^	43.85 (7)	C4—C3—C2	121.1 (3)
O6—K1—O5^ii^	71.21 (6)	C5—C4—C3	120.9 (3)
S1^ii^—K1—O5^ii^	24.25 (4)	C5—C4—H4	119.5
O3^iv^—K1—K1^vi^	111.17 (5)	C3—C4—H4	119.5
O7^iii^—K1—K1^vi^	109.12 (6)	O2—C5—C4	119.4 (3)
O5^i^—K1—K1^vi^	162.75 (6)	O2—C5—C6	119.7 (3)
O6^ii^—K1—K1^vi^	79.25 (6)	C4—C5—C6	120.9 (3)
O6—K1—K1^vi^	35.35 (5)	C1—C6—C5	116.8 (3)
S1^ii^—K1—K1^vi^	60.42 (2)	C1—C6—C7	120.2 (3)
O5^ii^—K1—K1^vi^	36.40 (4)	C5—C6—C7	123.0 (3)
O3^iv^—K1—K1^ii^	117.97 (5)	O3—C7—C8	122.4 (3)
O7^iii^—K1—K1^ii^	90.78 (8)	O3—C7—C6	121.6 (3)
O5^i^—K1—K1^ii^	49.41 (6)	C8—C7—C6	116.0 (3)
O6^ii^—K1—K1^ii^	35.37 (6)	C9—C8—C7	121.4 (3)
O6—K1—K1^ii^	146.71 (6)	C9—C8—H8	119.3
S1^ii^—K1—K1^ii^	53.48 (2)	C7—C8—H8	119.3
O5^ii^—K1—K1^ii^	77.10 (4)	C8—C9—O4	121.6 (3)
K1^vi^—K1—K1^ii^	113.47 (3)	C8—C9—C10	127.9 (3)
O3^iv^—K1—K1^viii^	160.42 (6)	O4—C9—C10	110.5 (3)
O7^iii^—K1—K1^viii^	49.44 (7)	C11—C10—C15	118.2 (3)
O5^i^—K1—K1^viii^	96.34 (5)	C11—C10—C9	121.0 (3)
O6^ii^—K1—K1^viii^	71.15 (7)	C15—C10—C9	120.8 (3)
O6—K1—K1^viii^	98.28 (6)	C10—C11—C12	121.9 (4)
S1^ii^—K1—K1^viii^	53.85 (2)	C10—C11—H11	119.1
O5^ii^—K1—K1^viii^	60.52 (4)	C12—C11—H11	119.1
K1^vi^—K1—K1^viii^	74.040 (15)	C13—C12—C11	119.4 (4)
K1^ii^—K1—K1^viii^	74.040 (15)	C13—C12—H12	120.3
O5—S1—O6	111.97 (16)	C11—C12—H12	120.3
O5—S1—O7	111.99 (17)	C12—C13—C14	120.7 (4)
O6—S1—O7	112.33 (19)	C12—C13—H13	119.6
O5—S1—C2	108.86 (15)	C14—C13—H13	119.6
O6—S1—C2	106.85 (15)	C13—C14—C15	119.5 (4)
O7—S1—C2	104.36 (16)	C13—C14—H14	120.3
O5—S1—K1^vi^	78.02 (11)	C15—C14—H14	120.3
O6—S1—K1^vi^	51.24 (10)	C14—C15—C10	120.3 (4)
O7—S1—K1^vi^	92.71 (12)	C14—C15—H15	119.8
C2—S1—K1^vi^	156.86 (12)	C10—C15—H15	119.8
C3—O1—H1	109.5		
			
O6—S1—O5—K1^v^	−89.8 (8)	O5—S1—C2—C3	−127.7 (3)
O7—S1—O5—K1^v^	37.4 (8)	O6—S1—C2—C3	111.2 (3)
C2—S1—O5—K1^v^	152.3 (7)	O7—S1—C2—C3	−8.0 (3)
K1^vi^—S1—O5—K1^v^	−50.7 (7)	K1^vi^—S1—C2—C3	128.4 (3)
O6—S1—O5—K1^vi^	−39.14 (14)	C1—C2—C3—O1	179.6 (3)
O7—S1—O5—K1^vi^	88.08 (15)	S1—C2—C3—O1	2.9 (5)
C2—S1—O5—K1^vi^	−157.05 (13)	C1—C2—C3—C4	0.4 (5)
O5—S1—O6—K1^vi^	52.35 (18)	S1—C2—C3—C4	−176.3 (3)
O7—S1—O6—K1^vi^	−74.68 (17)	O1—C3—C4—C5	−179.4 (3)
C2—S1—O6—K1^vi^	171.45 (13)	C2—C3—C4—C5	−0.1 (5)
O5—S1—O6—K1	175.02 (14)	C3—C4—C5—O2	178.0 (3)
O7—S1—O6—K1	48.0 (2)	C3—C4—C5—C6	−0.8 (5)
C2—S1—O6—K1	−65.88 (19)	O4—C1—C6—C5	177.3 (3)
K1^vi^—S1—O6—K1	122.7 (2)	C2—C1—C6—C5	−1.0 (4)
O3^iv^—K1—O6—S1	86.63 (17)	O4—C1—C6—C7	−1.9 (4)
O7^iii^—K1—O6—S1	−23.78 (19)	C2—C1—C6—C7	179.7 (3)
O5^i^—K1—O6—S1	68.3 (4)	O2—C5—C6—C1	−177.4 (3)
O6^ii^—K1—O6—S1	−146.56 (11)	C4—C5—C6—C1	1.3 (5)
S1^ii^—K1—O6—S1	−127.83 (16)	O2—C5—C6—C7	1.8 (5)
O5^ii^—K1—O6—S1	−128.54 (18)	C4—C5—C6—C7	−179.5 (3)
K1^vi^—K1—O6—S1	−120.4 (2)	K1^iv^—O3—C7—C8	−19.5 (6)
K1^ii^—K1—O6—S1	−147.10 (9)	K1^iv^—O3—C7—C6	161.6 (3)
K1^viii^—K1—O6—S1	−73.63 (16)	C1—C6—C7—O3	179.0 (3)
O3^iv^—K1—O6—K1^vi^	−153.01 (12)	C5—C6—C7—O3	−0.2 (5)
O7^iii^—K1—O6—K1^vi^	96.58 (12)	C1—C6—C7—C8	0.0 (4)
O5^i^—K1—O6—K1^vi^	−171.4 (2)	C5—C6—C7—C8	−179.2 (3)
O6^ii^—K1—O6—K1^vi^	−26.21 (19)	O3—C7—C8—C9	−177.0 (3)
S1^ii^—K1—O6—K1^vi^	−7.48 (11)	C6—C7—C8—C9	1.9 (5)
O5^ii^—K1—O6—K1^vi^	−8.18 (9)	C7—C8—C9—O4	−2.0 (5)
K1^ii^—K1—O6—K1^vi^	−26.7 (2)	C7—C8—C9—C10	179.1 (3)
K1^viii^—K1—O6—K1^vi^	46.72 (11)	C1—O4—C9—C8	0.1 (4)
O5—S1—O7—K1^vii^	−24.2 (5)	C1—O4—C9—C10	179.1 (2)
O6—S1—O7—K1^vii^	102.8 (5)	C8—C9—C10—C11	−8.7 (5)
C2—S1—O7—K1^vii^	−141.8 (4)	O4—C9—C10—C11	172.3 (3)
K1^vi^—S1—O7—K1^vii^	54.0 (5)	C8—C9—C10—C15	171.8 (3)
C9—O4—C1—C6	1.9 (4)	O4—C9—C10—C15	−7.2 (4)
C9—O4—C1—C2	−179.6 (2)	C15—C10—C11—C12	−1.1 (6)
O4—C1—C2—C3	−178.2 (3)	C9—C10—C11—C12	179.5 (4)
C6—C1—C2—C3	0.2 (5)	C10—C11—C12—C13	−0.5 (8)
O4—C1—C2—S1	−1.4 (4)	C11—C12—C13—C14	1.6 (8)
C6—C1—C2—S1	177.0 (2)	C12—C13—C14—C15	−1.0 (7)
O5—S1—C2—C1	55.8 (3)	C13—C14—C15—C10	−0.6 (6)
O6—S1—C2—C1	−65.3 (3)	C11—C10—C15—C14	1.7 (5)
O7—S1—C2—C1	175.5 (3)	C9—C10—C15—C14	−178.9 (3)
K1^vi^—S1—C2—C1	−48.2 (4)		

Symmetry codes: (i) *x*, *y*, *z*+1; (ii) *x*, −*y*+1/2, *z*+1/2; (iii) −*x*+1/2, *y*, *z*+1/2; (iv) −*x*, −*y*+1, −*z*+1; (v) *x*, *y*, *z*−1; (vi) *x*, −*y*+1/2, *z*−1/2; (vii) −*x*+1/2, *y*, *z*−1/2; (viii) −*x*+1/2, −*y*+1/2, *z*.

### Hydrogen-bond geometry (Å, °)

**Table:**

*D*—H···*A*	*D*—H	H···*A*	*D*···*A*	*D*—H···*A*
O1—H1···O7	0.82	1.84	2.588 (4)	152
O2—H2···O3	0.82	1.87	2.604 (4)	148
